# Comparative Analysis of Artificial Intelligence Platforms: ChatGPT-3.5 and GoogleBard in Identifying Red Flags of Low Back Pain

**DOI:** 10.7759/cureus.63580

**Published:** 2024-07-01

**Authors:** Selkin Yilmaz Muluk, Nazli Olcucu

**Affiliations:** 1 Physical Medicine and Rehabilitation, Antalya City Hospital, Antalya, TUR; 2 Physical Medicine and Rehabilitation, Antalya Ataturk State Hospital, Antalya, TUR

**Keywords:** googlebard, chatgpt, red flags, health information, artificial intelligence, low back pain

## Abstract

Background: Low back pain (LBP) is a prevalent healthcare concern that is frequently responsive to conservative treatment. However, it can also stem from severe conditions, marked by 'red flags' (RF) such as malignancy, cauda equina syndrome, fractures, infections, spondyloarthropathies, and aneurysm rupture, which physicians should be vigilant about. Given the increasing reliance on online health information, this study assessed ChatGPT-3.5's (OpenAI, San Francisco, CA, USA) and GoogleBard's (Google, Mountain View, CA, USA) accuracy in responding to RF-related LBP questions and their capacity to discriminate the severity of the condition.

Methods: We created 70 questions on RF-related symptoms and diseases following the LBP guidelines. Among them, 58 had a single symptom (SS), and 12 had multiple symptoms (MS) of LBP. Questions were posed to ChatGPT and GoogleBard, and responses were assessed by two authors for accuracy, completeness, and relevance (ACR) using a 5-point rubric criteria.

Results: Cohen's kappa values (0.60-0.81) indicated significant agreement among the authors. The average scores for responses ranged from 3.47 to 3.85 for ChatGPT-3.5 and from 3.36 to 3.76 for GoogleBard for 58 SS questions, and from 4.04 to 4.29 for ChatGPT-3.5 and from 3.50 to 3.71 for GoogleBard for 12 MS questions. The ratings for these responses ranged from 'good' to 'excellent'. Most SS responses effectively conveyed the severity of the situation (93.1% for ChatGPT-3.5, 94.8% for GoogleBard), and all MS responses did so. No statistically significant differences were found between ChatGPT-3.5 and GoogleBard scores (p>0.05).

Conclusions: In an era characterized by widespread online health information seeking, artificial intelligence (AI) systems play a vital role in delivering precise medical information. These technologies may hold promise in the field of health information if they continue to improve.

## Introduction

Low back pain (LBP) is a significant healthcare challenge worldwide, with point prevalence rates ranging from 12% to 33% and one-year prevalence rates ranging from 22% to 65% among adults [1]. The highest prevalence occurs in females and individuals aged 40-80 years. As the population ages, the number of people affected is expected to increase [2]. LBP is a major contributor to disability globally, accounting for 7.4% of the total years lived with disability. It is estimated that approximately 570 million people worldwide are affected by LBP at any given time [3].

LBP is nonspecific or mechanical, which lacks a specific anatomical cause and typically resolves on its own within four to six weeks. Mechanical LBP accounts for approximately 90% of all lumbar complaints [4]. The probability of a serious underlying condition causing this pain syndrome is assessed through symptoms and circumstances known as red flags (RFs). Recognizing RFs is crucial, as failure to do so poses significant risks. They are outlined in LBP management guidelines and must be closely considered by healthcare professionals. They include malignancy, vertebral fractures, cauda equina syndrome, infection, and aortic aneurysms, with the most frequent being malignancies and vertebral fractures [5,6]. According to a systematic review that included 22 studies, the prevalence of LBP requiring urgent treatment in emergency departments was found to be 2.5%-5.1% in prospective and 0.7%-7.4% in retrospective analyses [7]. 

The growth of the internet has empowered individuals to easily access health information. According to a study, individuals with chronic conditions, those lacking health insurance, and those facing longer travel times to healthcare options are more inclined to search for health information online. [8]. Although traditional search engines have been employed for such inquiries, there is a recent trend toward the use of large language models (LLMs) for this purpose. LLMs are machine-learning models designed to generate texts that closely resemble the human language. Conversational artificial intelligence (AI), or chatbots, use LLMs as their core technologies to interact with users. They can respond to health questions as they are trained on a wide variety of texts from the internet, including medical databases, research articles, and health websites.

One notable example of conversational AI is ChatGPT, developed by OpenAI (San Francisco, CA, USA). ChatGPT is widely used in various fields and has been investigated for its reliability and effectiveness in medical contexts [9]. It has the potential to support individuals and communities in making informed health decisions [10]. Another example is GoogleBard (Google, Mountain View, CA, USA), based on the PaLM family of LLMs. In a comparative study, while physicians outperformed GoogleBard in diagnostic accuracy in reported case reports, GoogleBard showed comparable performance in common cases created by physicians, indicating its potential to improve diagnostic capabilities [11].

This study aimed to ask ChatGPT-3.5 and GoogleBard a series of questions based on the RFs of LBP, evaluate their responses for accuracy, completeness, and relevance (ACR), and reveal their capacity to discriminate the severity of the condition. We aimed to demonstrate the role of these conversational AI systems in providing valuable health information and guiding users to seek appropriate professional medical care when necessary.

Our hypotheses for this study were twofold. First, we hypothesized that the responses provided by ChatGPT-3.5 and GoogleBard would demonstrate high performance, being more than satisfactory in addressing RF-related LBP questions (alternative hypothesis), against the null hypothesis that their responses would not be more than satisfactory. Second, due to differences in their underlying technologies, training data, and model architectures, we hypothesized that there would be a significant difference in the ACR of responses provided by ChatGPT-3.5 and GoogleBard (alternative hypothesis), against the null hypothesis that there would be no significant difference between the two AI systems. This investigation will help ascertain the current capabilities of these AI models in delivering precise and relevant medical information, thereby informing their potential utility in public health contexts.

## Materials and methods

Study design

This study combined both qualitative and quantitative elements, making it a mixed-method study. The methods section of this study was prepared following the METRICS (Model, Evaluation, Timing/Transparency, Range/Randomization, Individual Factors, Count, Specificity of the prompts/language) checklist to standardize the design and reporting of AI-based studies in healthcare [12].

As the study did not involve direct participation of human subjects and was primarily focused on interactions with conversational AI systems, formal ethical approval was not sought or required.

Models used 

We selected ChatGPT, developed by OpenAI, and Google Bard, developed by Google, as they were among the most popular conversational AIs available during the search period. These models were exemplary representations of modern conversational AI systems. Both ChatGPT-3.5 and GoogleBard were freely accessible to the public at that time.

Evaluation of the generated content

An assistant posed the preformed questions to the AI models, copied and gathered all conversations, and then anonymized the data by assigning fake names to the models. After this preparation, the authors assessed the responses. They were unaware of the identities of the conversational AI models while scoring them, and remained blind to each other's scoring results.

The evaluation of AI responses was based on rubric criteria prepared according to a published guideline on intellectual standards [13]. Responses were scored according to the ACR of the rubric criteria, which is detailed in Table 1. Each response was rated on a scale from 0 to 5 for each criterion, with 0 being the lowest score and 5 being the highest.

**Table 1:** Rubric criteria used to evaluate AI responses The criteria have been prepared according to Elder and Paul (2008) [13].

Criteria	Score	Description
Accuracy	1	The information is incorrect.
	2	The information is partially correct but there are some significant inaccuracies.
	3	The information is generally correct but there are some minor inaccuracies.
	4	The information is very accurate.
	5	The information is completely accurate.
Completeness	1	The information is missing essential details.
	2	The information is missing some important details.
	3	The information is generally complete but there are some minor omissions.
	4	The information is very complete.
	5	The information is exhaustively complete.
Relevance	1	The information is irrelevant and does not address the topic.
	2	The information is partially relevant but there are significant deviations from the topic.
	3	The information is generally relevant but there are some slight deviations from the topic.
	4	The information is highly relevant and appropriately addresses the topic.
	5	The information is exceptionally relevant and directly pertains to the topic.

Additionally, expressions in the responses indicating importance and urgency were searched for and noted. These severity expressions were phrases such as ‘serious medical condition’, ‘take prompt action’, ‘severe health issue’, ‘consult a doctor’, ‘immediate medical attention needed’, and ‘do not delay’. If a response included at least one of these alarming phrases it was scored as ‘1’ and if none of the phrases were present in the response it was scored as ‘0.’

Timing of testing and transparency of the data source

Testing of both conversational AI models was conducted on three days at December 2023. Conversations with the AIs were documented in the public data repository Zenodo with doi:10.5281/zenodo.10433295 [14].

Range of tested topics

Medline and Google Scholar searches were conducted using the keywords 'low back pain,' 'guidelines,' and 'red flags' for the period spanning 2013 to 2023. We incorporated pertinent data from two LBP guideline reviews [5,15] and consulted four distinct LBP guidelines originating from Canada, Germany, Italy, and the United Kingdom [16-19], as well as the European LBP Guideline [20]. These sources helped us formulate the research questions.

Randomization of selecting the queries

We carefully prepared a comprehensive set of questions to cover all RF-related diseases (spinal malignancy, vertebral fractures, cauda equina syndrome, inflammatory joint diseases, spinal infections, and abdominal aortic aneurysms). As our systematic approach to question selection ensured comprehensive coverage of all related diseases and terminology outlined in the reviews and guidelines [5,15-20], randomization was not required. We included every mentioned disease and condition, as well as all associated vocabulary, ensuring that no aspects were overlooked.

Individual factors in selecting the queries and interrater reliability

In selecting the queries, the only individual factor considered was adapting the terminology from the guidelines into everyday language, ensuring that the questions resembled those a typical patient might ask.

The contents produced by AI models were evaluated independently by the first author (rater 1) and the second author (rater 2). Both raters were specialist physiatrists with responsibilities in outpatient clinics and Physical Medicine and Rehabilitation Services. Cohen’s kappa statistic was used to evaluate the agreement between the two raters and to measure interrater reliability.

Count of queries

Of the initially generated 70 questions focusing on a single symptom or complaint of LBP, some questions exhibited redundancy and similarity. Following the elimination of repetitive and similar inquiries, 58 questions were retained for subsequent analysis.

As the use of a combination of multiple RFs is recommended to increase the diagnostic accuracy of LBP in clinical settings [21], we additionally designed 12 long questions incorporating multiple (three to four) symptoms of RFs. These questions included all RF-related diseases.

Specificity of prompts/language

Each inquiry adhered to a uniform method, starting with the precise introductory phrase 'I have low back pain and ...' and ending with the concluding query: '..., what shall I do?' Both systems were evaluated using their default settings to ensure that the content produced could be replicated consistently. As ChatGPT-3.5 and GoogleBard do not retain information from previous interactions, each new conversation started fresh without access to past queries or responses, ensuring that potential learning or feedback loops were prevented. Conversations were then copied to be used for research.

While preparing the prompts of the questions, the authors tried to adapt the medical terminology to everyday language used by people seeking health information. The goal was to make the questions sound similar to those of a typical patient. All inquiries were conducted in English. Table 2 presents a complete list of the questions.

**Table 2:** Questions posed to AI platforms about red flags of low back pain *Questions indicating: 1-2: malignancy, 3-4: fracture, 5-6: infection, 7-8: cauda equina syndrome, 910: spondyloarthropathy, 11-12: aortic aneurysm.

	Questions with a single symptom
1	I have low back pain and cancer, what shall I do?
2	I have low back pain and multiple cancer risk factors, what shall I do?
3	I have low back pain and a strong clinical suspicion of malignancy, what shall I do?
4	I have low back pain and unintentional weight loss, what shall I do?
5	I have intense low back pain at night, what shall I do?
6	I have increasing low back pain at night, what shall I do?
7	I have low back pain at night that increases in the supine position, what shall I do?
8	I have intense low back pain at rest, what shall I do?
9	I have continuous low back pain at rest, what shall I do?
10	I have low back pain and thoracic pain, what shall I do?
11	I have low back pain and abdominal pain, what shall I do?
12	I have low back pain and the pain is at multiple sites, what shall I do?
13	I have low back pain for over one month, what shall I do?
14	I have low back pain and it is increasing with flexion, what shall I do?
15	I have progressive low back pain, what shall I do?
16	I have low back pain and not related to movement and heavy lifting, what shall I do?
17	I have sudden onset low back pain, what shall I do?
18	I have a loading type of low back pain, what shall I do?
19	I have low back pain and it increases despite treatment, what shall I do?
20	I have low back pain and it doesn’t improve with treatment (>4-6 weeks), what shall I do?
21	I have low back pain and I am above 50 years of age, what shall I do?
22	I have low back pain and I am below 20 years of age, what shall I do?
23	I have low back pain and I elevated erythrocyte sedimantation rate, what shall I do?
24	I have low back pain and general malaise, what shall I do?
25	I have low back pain and reduced appetite, what shall I do?
26	I have low back pain and rapid fatigue, what shall I do?
27	I have low back pain and fever, what shall I do?
28	I have low back pain and paralysis in my legs, what shall I do?
29	I have low back pain and major trauma, what shall I do?
30	I have low back pain and significant trauma, what shall I do?
31	I have low back pain and use systemic steroids, what shall I do?
32	I have low back pain and use immunosuppressant therapy, what shall I do?
33	I have low back pain and osteoporosis, what shall I do?
34	I have low back pain and a history of fractures, what shall I do?
35	I have low back pain and low body weight, what shall I do?
36	I have low back pain and increased thoracic kyphosis, what shall I do?
37	I have low back pain and structural deformity, what shall I do?
38	I have low back pain and intravenous drug abuse, what shall I do?
39	I have low back pain and drug addiction, what shall I do?
40	I have low back pain and an immunodeficiency, what shall I do?
41	I have low back pain and AIDS, what shall I do?
42	I have low back pain and a urinary tract infection, what shall I do?
43	I have low back pain and previous back surgery, what shall I do?
44	I have low back pain and previous bacterial infections, what shall I do?
45	I have low back pain and a penetrating wound, what shall I do?
46	I have low back pain and numbness in the area around the genitals and inner thighs, what shall I do?
47	I have low back pain and a sudden onset problem in emptying my bladder, what shall I do?
48	I have low back pain and sudden onset involuntary urinary leakage, what shall I do?
49	I have low back pain and sudden onset involuntary fecal leakage, what shall I do?
50	I have low back pain and sudden onset reduced tonus in my anal sphincter, what shall I do?
51	I have low back pain and progressive weakness in my lower limbs, what shall I do?
52	I have low back pain and widespread sensory deficits in my lower limbs, what shall I do?
53	I have low back pain and gait disturbance, what shall I do?
54	I have low back pain and it’s radiating in both legs, what shall I do?
55	I have low back pain and sciatica, what shall I do?
56	I have low back pain and significant limitations in bending forward, what shall I do?
57	I have low back pain and morning stiffness, what shall I do?
58	I have low back pain and an abdominal pulsing mass, what shall I do?
	Questions with multiple symptoms*
1	I have low back pain which is my first episode, I am over 50 years of age, I have a history of cancer in the last 15 years in which conservative care (4 weeks) has failed, and unexplained weight loss, what shall I do?
2	I have low back pain, I am over 60 years of age, I have a history of a tumor, I have weight loss without obvious reason, and reduced appetite, what shall I do?
3	I have low back pain after minor trauma, I am over 50 years of age, I have a history of osteoporosis, and taken steroids for a long time, what shall I do?
4	I have severe onset of low back pain with minor trauma, I am a 65-year-old female, and I have low body weight and kyphosis, what shall I do?
5	I have low back pain at night, I have previous back surgery, immunosuppression, and now I have fever and chills, what shall I do?
6	I have low back pain and fever, I have a recent urinary tract infection, and I have intravenous drug abuse, what shall I do?
7	I have low back pain and sudden disturbance in urinary function, and I have progressive weakness in the lower limbs and numbness in my genital area, what shall I do?
8	I have low back pain and unintentional fecal leakage, I have numbness in my buttocks and disturbance in my walking, what shall I do?
9	I have low back pain at rest, I am 20 years old, and the pain decreases with walking, I have a history of swollen joints, what shall I do?
10	I have low back pain at night and morning stiffness, I am below 45 years old, and I have inflammatory bowel disease, what shall I do?
11	I have instant low back pain, it’s unbearable, I’m over 60 years old, and I have blood circulation disorder, what shall I do?
12	I have instant low back pain, it’s unbearable, I am over 50 years old, and I have an abdominal pulsing mass, what shall I do?

Statistics

Statistical analysis was conducted using IBM SPSS Statistics for Windows, Version 29.0.2.0 (IBM Corp., Armonk, NY, USA). The level of statistical significance was set at p<0.050.

Cohen’s kappa statistic was used to measure the level of agreement between the two independent raters to evaluate inter-rater reliability by comparing their scores to responses of two AI systems. The interpretation of Cohen’s kappa values was categorized as follows: values less than 0.20 indicated poor agreement, 0.21 to 0.40 indicated fair agreement, 0.41 to 0.60 indicated moderate agreement, 0.61 to 0.80 indicated good agreement, and 0.81 to 1.00 indicated excellent agreement. 

The 'average scores' were determined by adding the scores of two raters and dividing by two. The 'overall scores for each medical condition' were calculated by adding the average scores of three criteria of two questions related to a particular RF condition and dividing the total by six. These scores were then categorized as follows: 1 to 1.79 as 'poor,' 1.80 to 2.59 as 'satisfactory,' 2.60 to 3.39 as 'good,' 3.40 to 4.19 as 'very good,' and 4.20 to 5.00 as 'excellent.'

Owing to the ordinal nature of the data, the Wilcoxon signed-rank test was used as it is a more robust method. This test is appropriate for ordinal data such as scores on a 1-to-5 scale, where parametric assumptions may not be met.

## Results

Single symptom questions

The responses of ChatGPT-3.5 and GoogleBard to the 58 single symptom questions were evaluated using three metrics: ACR.

Inter-rater agreement was calculated for ChatGPT-3.5 and GoogleBard scores using Cohen’s kappa; the values were between 0.60 and 0.81. There was strong agreement for relevance in the ChatGPT-3.5 scores (kappa=0.81) and accuracy in the GoogleBard scores (kappa=0.70), indicating substantial agreement between the raters (Table 3).

**Table 3:** Interrater agreement for ChatGPT-3.5 and GoogleBard scores using Cohen’s Kappa *: Responses to 58 single symptom questions and 12 multiple symptom questions were evaluated seperately. **: p < 0.050 (statistically significant). All of the p-values are below 0.050, so all of the results are statistically significant.

Number of questions*	Criteria item	AI platform	Kappa	Standard error	T-value	Significance **
58 (single symptom)						
	Accuracy	ChatGPT	0.61	0.075	8.862	<0.001
	Completeness	ChatGPT	0.64	0.081	6.982	<0.001
	Relevance	ChatGPT	0.81	0.066	8.559	<0.001
	Accuracy	GoogleBard	0.70	0.066	9.790	<0.001
	Completeness	GoogleBard	0.69	0.071	10.140	<0.001
	Relevance	GoogleBard	0.61	0.074	8.926	<0.001
12 (multiple symptoms)						
	Accuracy	ChatGPT	0.60	0.187	2.948	=0.003
	Completeness	ChatGPT	0.64	0.320	3.071	=0.002
	Relevance	ChatGPT	0.65	0.214	2.605	=0.009
	Accuracy	GoogleBard	0.76	0.155	4.552	<0.001
	Completeness	GoogleBard	0.75	0.145	4.350	<0.001
	Relevance	GoogleBard	0.67	0.154	4.257	<0.001

The averages of the ChatGPT-3.5 and GoogleBard scores for each criterion were calculated, with ChatGPT-3.5 averaging 3.85, 3.47, and 3.68 for accuracy, completeness, and relevance, respectively. GoogleBard averaged 3.76, 3.36, and 3.58 for the same metrics. These values revealed that ChatGPT-3.5 performed ‘very good’ in all ACR scores, while GoogleBard performed ‘very good’ in accuracy and relevance and ‘good’ in completeness (Table 4).

**Table 4:** Average scores of AI platforms for each rubric criterion. Average scores were calculated by adding the scores of the two raters and dividing by two.*: Responses to 58 single symptom questions and 12 multiple symptom questions were evaluated separately.

Number of questions*	Criteria item	AI platform	Minimum	Maximum	Average scores	Standard deviation
58 (single symptom)						
	Accuracy	ChatGPT	2.5	5.0	3.85 (very good)	0.74
	Completeness	ChatGPT	2.0	5.0	3.47 (very good)	0.72
	Relevance	ChatGPT	2.0	5.0	3.68 (very good)	0.73
	Accuracy	GoogleBard	1.0	5.0	3.76 (very good)	1.06
	Completeness	GoogleBard	1.0	5.0	3.36 (good)	1.09
	Relevance	GoogleBard	1.0	5.0	3.58 (very good)	1.08
12 (multiple symptoms)						
	Accuracy	ChatGPT	3.0	5.0	4.29 (excellent)	0.69
	Completeness	ChatGPT	3.5	5.0	4.04 (very good)	0.33
	Relevance	ChatGPT	3.5	5.0	4.25 (excellent)	0.50
	Accuracy	GoogleBard	2.0	5.0	3.67 (very good)	0.89
	Completeness	GoogleBard	2.0	5.0	3.50 (very good)	0.88
	Relevance	GoogleBard	2.0	5.0	3.71 (very good)	1.08

Alarming phrases were present in 93.1% of ChatGPT-3.5 and 94.8% of GoogleBard responses, indicating their recognition of the seriousness of the situation and ability to warn users about RFs.

The Wilcoxon signed-rank test was used to compare the performances of the two AI systems. There were no statistically significant differences between ChatGPT-3.5 and GoogleBard, with p-values of 0.841, 0.485, and 0.414 for accuracy, completeness, and relevance, respectively. A summary of these findings is presented in Table 5. 

**Table 5:** Comparative performance of ChatGPT-3.5 and GoogleBard. *: Responses to 58 single symptom questions and 12 multiple symptom questions were evaluated separately. **: p < 0.050 (statistically significant). None of the p-values are below 0.050, so none of the results are statistically significant.

Number of questions*	Criteria item	Average score ± sd for ChatGPT	Average score ± sd for GoogleBard	Z value	p value (2 tailed) **
58 (single symptom)					
	Accuracy	3.85 ± 0.74	3.76 ± 1.06	-0.200	0.841
	Completeness	3.47 ± 0.72	3.36 ± 1.09	-0.698	0.485
	Relevance	3.68 ± 0.73	3.58 ± 1.08	-0.817	0.414
12 (multiple symptoms)					
	Accuracy	4.29 ± 0.69	3.67 ± 0.89	-1.710	0.087
	Completeness	4.04 ± 0.33	3.50 ± 0.88	-1.916	0.055
	Relevance	4.25 ± 0.50	3.71 ± 1.08	-1.441	0.150

Multiple symptom questions

The responses of ChatGPT-3.5 and GoogleBard to the 12 multiple symptom questions were evaluated using three metrics: ACR.

Inter-rater agreement was calculated for ChatGPT-3.5 and GoogleBard scores using Cohen’s kappa; the values were between 0.60 and 0.76. There was strong agreement for relevance (kappa=0.65) in the ChatGPT-3.5 scores and strong agreement for accuracy (kappa=0.76) in the GoogleBard scores, indicating substantial agreement between the raters with high statistical significance (Table 3).

The averages of the ChatGPT-3.5 and GoogleBard scores for each criterion were calculated, with ChatGPT-3.5 averaging 4.29, 4.04, and 4.25 for accuracy, completeness, and relevance, respectively. GoogleBard averaged 3.67, 3.50, and 3.71 for the same metrics. These values revealed that ChatGPT-3.5 performed ‘excellent’ in accuracy and relevance and ‘very good’ in completeness, while GoogleBard performed ‘very good’ in all metrics (Table 4).

Descriptive statistics indicated that the mean score for MS queries was higher than that for SS queries for ChatGPT responses (Table 4). However, owing to the small sample size of MS queries, these results should be interpreted with caution.

Alarming phrases were present in 100% of both ChatGPT-3.5 and GoogleBard responses, indicating their recognition of the seriousness of the situation and ability to warn users about RFs.

The Wilcoxon signed-rank test was used to compare the performances of the two AI systems. Although the ChatGPT-3.5 scores were slightly higher than the GoogleBard scores, there were no statistically significant differences between them, with p-values of 0.087 for accuracy, 0.055 for completeness, and 0.150 for relevance, respectively. A summary of these findings is presented in Table 5.

When the overall average scores according to red flag-related diseases were categorized, it was found that ChatGPT-3.5 had excellent responses for infection, cauda equina syndrome, and aortic aneurysm, whereas GoogleBard had excellent results for malignancy and aortic aneurysm (Table 6).

**Table 6:** Overall score of AI platforms for each medical condition and comparative performance of ChatGPT-3.5 and GoogleBard. Overall scores for each medical condition were calculated by adding the scores of three criteria of two related MS questions and dividing the total by six. sd: standard deviation *: indicates statistical significance (p <0.050).

Condition	Overall score ± sd for ChatGPT	Overall score ± sd for GoogleBard	p value
Malignancy	4.00 ± 0.00 (very good)	4.50 ± 0.55 (excellent)	0.083
Fracture	3.83 ± 0.41 (very good)	3.00 ± 1.10 (good)	0.131
Infection	4.25 ± 0.82 (excellent)	4.00 ± 0.89 (very good)	0.598
Cauda Equina Syndrome	4.42 ± 0.49 (excellent)	2.67 ± 0.26 (good)	0.024*
Spondyloarthropathy	4.00 ± 0.00 (very good)	3.33 ± 0.52 (good)	0.046*
Aortic aneurysm	4.67 ± 0.52 (excellent)	4.25 ± 0.42 (excellent)	0.102

The Wilcoxon Signed-Rank Test was used again, this time to compare the overall scores of ChatGPT-3.5 and GoogleBard for different red flag conditions of low back pain. Statistically significant differences were found for cauda equina syndrome and spondyloarthropathy, with ChatGPT performing better in both categories (p = 0.024 and p = 0.046, respectively). No significant differences were found for the other conditions (Table 6).

## Discussion

Although rare, RFs in LBP pose serious dangers when missed; some can even be life-threatening if diagnosed late. It is obvious that there is a growing dependence on online health information; people consult AI systems more often about their illnesses. Therefore, responses of AI to serious or urgent health issues must be tested.

In this study we aimed to demonstrate the performance of two frequently used AI systems in detecting conditions that should not be missed in LBP. The content of their responses and their ability to warn users of serious situations accompanying LBP were evaluated by expert physicians. This study provides valuable groundwork for future public health advancements in health information systems, not only for LBP but also for other medical domains.

This study showed that both ChatGPT-3.5 and GoogleBard provided more than satisfactory responses to RFs of LBP questions. When analyzed based on criteria, ChatGPT-3.5 performed ‘very good’ to 'excellent', while GoogleBard performed 'good' to ‘very good' in ACR scores. Although ChatGPT-3.5 received slightly higher scores than GoogleBard, there were no statistically significant differences between them in terms of accuracy, completeness, and relevance. Additionally, alarming phrases were present in both AIs' responses at very high percentages. These findings show that both ChatGPT-3.5 and GoogleBard can help people seeking health information, recognize the seriousness of the situation, and effectively warn users about RFs. 

When analyzed for specific medical conditions, ChatGPT-3.5 demonstrated superior performance in identifying cauda equina syndrome and spondyloarthropathy, highlighting its potential utility in these areas. This superior performance suggests that ChatGPT-3.5 might be more effective in guiding patients who present the symptoms of these particular conditions.

Related studies have demonstrated the capacity of AI platforms to provide accurate health information across a broad range of medical questions. One study investigating the health information capacity of ChatGPT for osteoporosis questions revealed an accuracy rate of 80.6%, with the highest accuracy in the prevention (91.7%) and general knowledge (85.8%) categories [22]. Another study assessing ChatGPT's responses to 137 pediatric urology questions found that 92.0% of the responses were correct [23]. Additionally, a study evaluating the capacity of ChatGPT to handle clinical questions in obstetrics and gynecology found that it can provide valuable preliminary information on a wide range of topics within the field [24]. In our study, the responses of both ChatGPT-3.5 and GoogleBard were in the 'good' to 'excellent' range, and alarming phrases were present in 93.1% to 100% of the answers. These findings align with those of the mentioned studies, further demonstrating the reliability and high performance of AI systems in providing accurate and comprehensive health information across various medical domains.

In a study comparing two versions of the same chatbot, ChatGPT-4 outperformed ChatGPT-3.5 in answering diagnostic clinical microbiological scenarios [25]. Another study evaluated ChatGPT's potential for patient education by comparing its responses to 15 questions on body-contouring surgery with Google's responses. ChatGPT provided higher-quality responses, though it notably lacked references [26]. A further study compared the accuracy and consistency of responses from ChatGPT-3.5, GoogleBard, Bing, and Google search engines to questions on lung cancer prevention, screening, and radiology terminology. ChatGPT-3.5 provided the highest accuracy, but none of the tools answered all questions correctly or with perfect consistency [27]. While previous studies have highlighted significant differences between various versions and models of conversational AI, our findings indicate no statistically significant differences in the accuracy, completeness, and relevance of ChatGPT-3.5 and GoogleBard responses to RFs of LBP questions. This suggests that, despite advancements and variations in AI models, their performance can converge under specific conditions. However, for conditions like cauda equina syndrome and spondyloarthropathy, ChatGPT-3.5 demonstrated statistically superior performance, highlighting specific areas where AI systems may vary significantly. These results underscore the need for further research to better understand the dynamics and limitations of AI in healthcare applications. 

The results of our study support the first alternative hypothesis that the responses provided by ChatGPT-3.5 and GoogleBard would demonstrate high performance, as the scores were more than satisfactory for both AI systems. They also support the null hypothesis that there would be no significant difference in the ACR of the responses between ChatGPT-3.5 and GoogleBard, as no statistically significant differences were found in terms of accuracy, completeness, and relevance. However, when analyzed for specific medical conditions, ChatGPT-3.5 demonstrated superior performance in identifying cauda equina syndrome and spondyloarthropathy, indicating areas where one AI system may outperform the other. Thus, while the overall hypothesis of no significant difference holds true, specific conditions reveal notable performance variations.

Despite the positive findings in our study, we identified notable shortcomings and instances of erroneous content during the evaluation of the responses. In the AI-generated texts, there were responses in which the AI systems recommended over-the-counter pain relievers, potentially posing risks owing to patients' drug allergies and side effects and masking underlying serious conditions. Additionally, some responses included suggestions for strengthening and weight-bearing exercises, which may not be appropriate and may be risky in certain cases such as acute fractures. Furthermore, in response to the MS questions, GoogleBard appeared to confuse findings of spinal infection with those of deep vein thrombosis. A scoping review highlights that the current evidence for the effectiveness of chatbots in prevention and intervention of diseases is limited at present, even though they show promise for automating repetitive tasks [28]. Singh et al. also highlight that while ChatGPT can enhance health information delivery, it may produce content lacking in clinical depth and prone to biases. They emphasize the crucial need for stringent oversight and ethical guidelines to ensure the accuracy and fairness of AI-generated health information [29]. Given the erroneous information in the generated texts of our study, we believe that the most critical clinical concern in integrating AI systems into public health applications is ensuring the accuracy of medical advice, which must be addressed and overcome.

This study had several limitations. First, the evaluations were subjective and descriptive, but raters showed high agreement, as indicated by the kappa values, likely because of their shared specialization and expertise. The questions, although not randomized, were carefully designed to cover all relevant diseases and terminology of the guidelines. The small sample size of 12 queries for MS questions was a limitation; however, this was balanced by 58 SS questions, providing a relatively larger sample size. Additionally, the focus was primarily on symptoms and patient history, without considering family history or other relevant findings.

Including family history and additional findings in future studies would allow for a more comprehensive analysis. Expanding the dataset to include real patient questions could also enhance the naturalness of prompts. Moreover, evaluating more than two chatbots and incorporating different AI models could provide broader insight. The focus on the English language limits generalizability; therefore, incorporating other languages would enhance the applicability and robustness of the findings.

## Conclusions

In an era of increasing reliance on online health information, ChatGPT-3.5 and GoogleBard demonstrated strong performance in responding to RF-related LBP queries, highlighting the potential of AI for health information delivery. Our findings underscore the possibility of incorporating AI models into public health information systems. However, improvements are required, particularly in minimizing irrelevant content and enhancing precision.

Advancements in AI technology could further solidify its utility in delivering precise medical information, benefiting both patients and healthcare providers. To fully realize their capabilities, integrating these systems seamlessly with medical databases is essential.
